# The effects of dietary net energy on grow-finish performance and carcass characteristics of male market pigs managed with immunological castration (Improvest)

**DOI:** 10.1093/tas/txae027

**Published:** 2024-03-01

**Authors:** Benjamin M Bohrer, Yifei Wang, Jose L Landero, Malachy Young, Blaine Hansen, D Steve Pollmann, Marnie A Mellencamp, Leanne Van De Weyer, Alvaro Aldaz

**Affiliations:** Department of Animal Sciences, The Ohio State University, Columbus, OH 43210, USA; Department of Animal Sciences, The Ohio State University, Columbus, OH 43210, USA; Gowan’s Feed Consulting, Wainwright, Alberta, CanadaT9W 1L2; Gowan’s Feed Consulting, Wainwright, Alberta, CanadaT9W 1L2; BCH Consulting LLC, Atlantic, IA, 50022, USA; DSP Consulting LLC, Alpine, UT, 84004, USA; Zoetis Inc., Parsippany, New Jersey, NJ 07054, USA; Zoetis Canada Inc., Kirkland, Quebec, CanadaH9H 4M7; Zoetis Inc., Parsippany, New Jersey, NJ 07054, USA

**Keywords:** immunological castration, immunocastration, anti-GnRF, pig production, dietary energy

## Abstract

The objective was to determine the effects of dietary net energy (**NE**) during the grow-finish period on live performance and carcass characteristics of intact male pigs managed with immunological castration (Improvest) compared with physically castrated (**PC**) male pigs. The 101-d study began when 1,008 pigs (504 intact male pigs and 504 PC male pigs; 10 wk old) were allocated by weight to 48 pens with 21 intact males or 21 PC males per pen. Three dietary NE treatments were fed to pigs using five dietary phases (dietary programs were formulated according to standardized ileal digestible lysine requirements of Improvest males or PC males) to provide an average of 2,212 kcal/kg (low NE), 2,337 kcal/kg (medium NE), or 2,462 kcal/kg (high NE). The experiment was designed and analyzed as a 2 × 3 factorial with main effects of Improvest management and NE. For the overall study period, there were no significant interactions between Improvest management and NE (*P* ≥ 0.19) for average daily feed intake (**ADFI**), average daily gain (**ADG**), or gain:feed (**G:F**). There were also no significant interactions between Improvest management and NE (*P* ≥ 0.06) for carcass characteristics. Improvest males consumed less feed (5.3% lower ADFI; *P* < 0.01), grew faster (5.1% greater ADG; *P* < 0.01), and were more efficient (11.2% greater G:F; *P* < 0.01) compared with PC males. Hot carcass weight (**HCW**) did not differ (*P* = 0.16) between Improvest males and PC males (attributed to 1.6 percentage unit lower dressing percentage for Improvest males; *P* < 0.01); however, Improvest males were leaner (0.9 mm less backfat and 0.65% greater predicted lean yield; *P* < 0.01) compared with PC males. For the overall study period, pigs fed low NE and medium NE diets consumed 7.5% and 4.6% more feed (*P* < 0.01) than pigs fed high NE diets, respectively, and pigs fed low NE diets grew 1.7% slower (*P *< 0.02) than pigs fed medium NE and high NE diets. This resulted in pigs fed low NE diets having 4.4% lower G:F compared with pigs fed medium NE and 8.6% lower G:F compared with pigs fed high NE diets (*P* < 0.01). Pigs fed low NE had 3.0 kg lighter (*P* < 0.01) HCW compared with medium NE, while high NE had intermediate HCW that did not differ from the other two treatments. Overall, typical Improvest response levels for live performance and carcass characteristics when compared with PC males were achieved for each of the NE treatments evaluated in this study.

## Teaser

This study compared the effect of dietary net energy (**NE** ) in grow-finish pig diets when male pigs were managed with physical castration or immunological castration. As a result of growing as intact male pigs for a significant portion of the grow-finish period, pigs managed with immunological castration had 11.2% greater feed efficiency compared with physically castrated male pigs. There were a limited number of statistically significant interactions in this study, indicating that typical response levels for important performance parameters can be sustained when feeding low NE diets to immunologically castrated male pigs.

## Introduction

Improvest (marketed under the trade name of Improvac, Innosure, or Vivax in some parts of the world; Zoetis Inc., Parsippany, NJ, USA) is a gonadotropin-releasing factor (GnRF) analog-diphtheria toxoid conjugate formulated product approved for the temporary suppression of testicular function and reduction of boar taint in intact male pigs intended for slaughter. Managing male pigs with Improvest has several secondary production advantages when compared with physically castrated (PC) male pigs, including 1 to 2 percentage unit improvement in preweaning livability, the management of ridgling pigs, 4% to 6% less feed consumption during the grow-finish period, 4% to 8% improvement in weight gain during the grow-finish period, 8% to 12% improvement in feed efficiency during the grow-finish period, and 1.0% to 1.5% unit improvement in cutting yields of merchandized meat cuts (Čandek-Potokar et al., 2017; Harsh et al., 2017; Needham et al., 2017; Poulsen Nautrup et al., 2018). Nutritional recommendations for male pigs managed with Improvest differ from those of PC males, which is attributed to differences in feed consumption and lean growth (Dunshea et al., 2013; Rueff et al., 2019). Specifically, it is recommended that levels of standardized ileal digestible (SID) lysine are elevated in diets for Improvest males during the pre-Improvest period when the pigs are growing/developing as intact male pigs. Recommendations for dietary energy levels for Improvest males have not been extensively researched to this point.

Energy is an important nutrient for growing animals and is the single most expensive component of swine diets on a total level of inclusion/consumption basis (Velayudhan et al., 2015; Noblet et al., 2022). Net energy (NE) of livestock diets is defined as the metabolizable energy of a diet after accounting for the heat of digestion, nutrient metabolism, and excretion of waste (Just, 1982; Zuidhof, 2019). The main source of energy found in North American swine diets is corn and/or corn by-products; however, some regions of the world where corn is not commonly grown, such as western Canada, eastern Europe, and southern Europe, may utilize other grains such as barley or wheat for the main source of energy in swine diets (Woyengo et al., 2014; Velayudhan et al., 2015). The energy content of corn (NE = 2,672 kcal/kg) is approximately 15% greater than that of barley (NE = 2,327 kcal/kg) and is approximately 8% greater than that of wheat (NE = 2,472 kcal/kg) (NRC, 2012). The NRC (2012) does not list dietary requirements for NE. The assumption for NE content of the diet in grow-finish pigs, which is presented as a reference in dietary requirement tables, was 2,475 kcal/kg. In field studies conducted by PIC, high-energy grow-finish diets comprised of corn and high concentrations of oils have been reported to have NE levels as high as 2,733 kcal/kg and low-energy grow-finish diets comprised of barley and low concentrations of oils have been reported to have NE levels as low as 2,116 kcal/kg (PIC, 2021). It is understandable that crops grown in the given region of the world where pigs are raised will dictate the ingredients available with which a nutritionist may formulate diets; however, the interaction of these formulation constraints with emerging technologies is often not fully elucidated.

To this point, investigation of the impacts of NE levels on live performance and carcass characteristics of market pigs is limited, even though several areas of the world feed pigs with ingredients with differing levels of NE. In particular, no current study exists comparing the effects of NE level for male pigs managed with immunological suppression of testicular function. Therefore, the purpose of this study was to determine the effects of NE level during the grow-finish period on live performance and carcass characteristics of intact male market pigs managed with immunological castration (Improvest) compared with PC male market pigs.

## Materials and Methods

The experiment was conducted at a commercial research grow-finish facility located in Alberta, Canada. Pigs were managed in accordance with the Canadian Council on Animal Care (2009).

### Animals

At the time of preweaning processing, male pigs (progeny from Duroc sires [PIC 800] × white line sows [PIC Camborough F1]; Genus PIC; Hendersonville, TN, USA) from three farrowing rooms at a commercial pig farm were selected for use in this study. Each farrowing room at the commercial sow barn consisted of 68 crates for a total of 750 to 800 pigs (males and females) per room. Within each litter of pigs, one-half of the male pigs were surgically castrated before 3 d of age according to NFACC (2014) guidelines, and the other half of the male pigs were left intact. At the time of weaning (21 d of age), all pigs from each farrowing room were transferred to a nursery facility (located approximately 0.75 km from the farrowing barn) where feeding, management, and stocking density were similar for all pigs. At approximately 10 wk of age, 504 PC males and 504 entire males were randomly selected from the nursery barn and transferred to the commercial research grow-finish facility. Following an acclimation period of 4 d, pigs were weighed and allotted to a total of eight replications, each replication consisting of six different dietary treatments (three of which were for PC males and three of which were for intact males). Each treatment within replication was randomly assigned to one of 48 single-sex pens (6.1 m × 2.4 m; 21 pigs/pen; initial stocking density of 0.7 m^2^/pig) in one room of the commercial grow-finish barn. Pens were equipped with a double-sided wet/dry stainless-steel feeder (Crystal Spring Hog Equipment; Agathe, Manitoba, Canada) and a single bowl drinker. The room was ventilated with negative pressure and was maintained within thermo-neutral temperatures for grow-finish pigs. Pigs had ad libitum access to feed and water throughout the duration of the grow-finish period.

### Administration of Improvest

Intact male pigs were administered Improvest (a GnRF analog-diphtheria toxoid conjugate product; Zoetis, Inc.) via injection by trained personnel according to manufacturer directions. The first Improvest dose was administered on day 29 of the study and the second Improvest dose (which triggers the temporary suppression of testicular function) was administered on day 57 of the study. In order to complete the federally mandated farm declaration documents, every intact male pig administered Improvest underwent a compliance check performed by Zoetis personnel (i.e., pigs were visually inspected for boar behavior a minimum of 14 d after the second dose and prior to any pigs being marketed; all suspect pigs who failed the compliance check were administered an additional dose of Improvest and an additional 14 d must have elapsed before they were marketed).

### Diets

**Figure 1.**
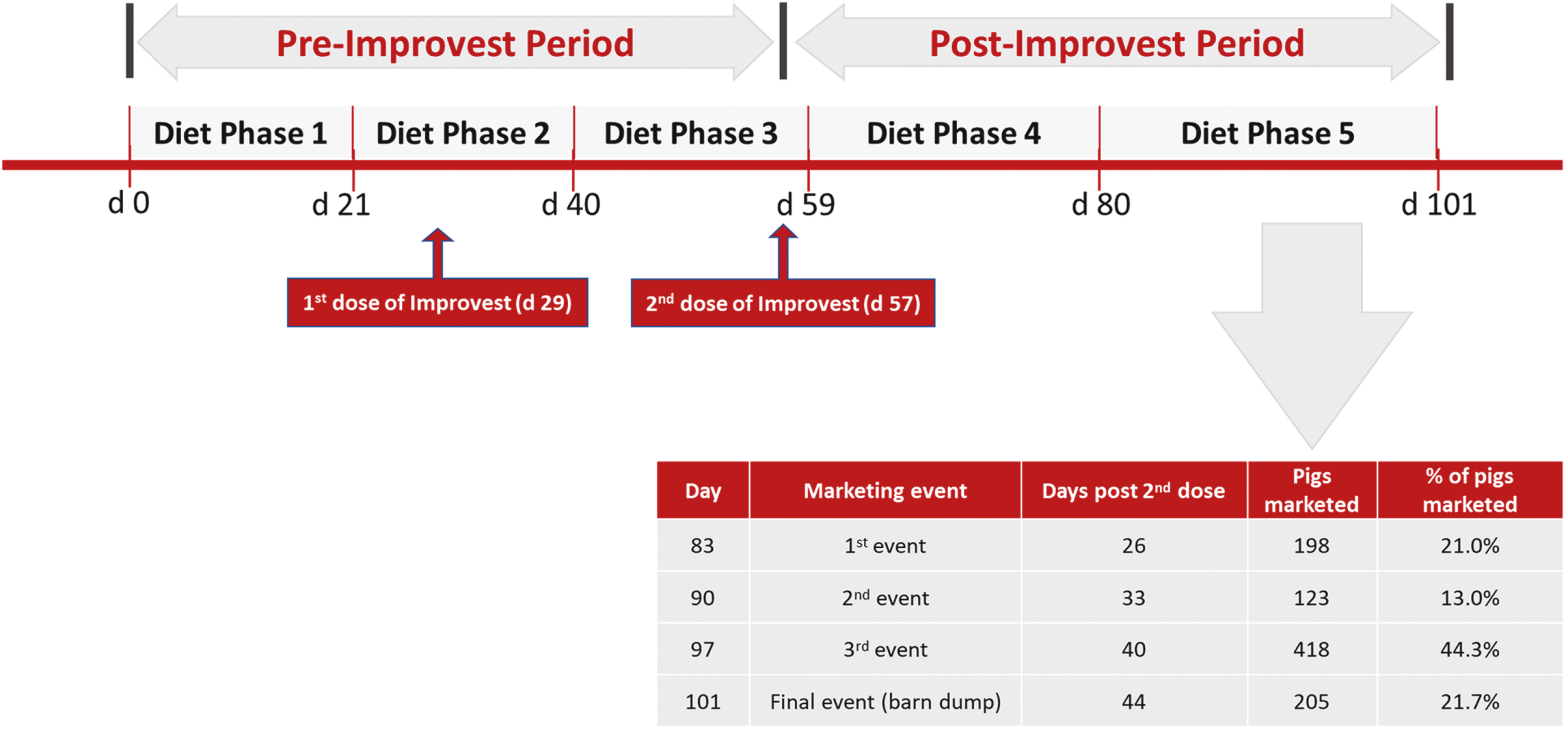
Timeline of study events.

The experiment was conducted using five dietary phases: phase 1 (days 0 to 21 of the study; targeted weights of 28 kg to 50 kg); phase 2 (days 22 to 40 of the study; targeted weights of 50 to 72 kg); phase 3 (days 41 to 59 of the study; targeted weights of 72 to 95 kg); phase 4 (days 60 to 80 of the study; targeted weights of 95 to 120 kg); phase 5 (day 81 of the study to marketing; targeted weights of 120 to 139 kg) (Figure 1). The second dose of Improvest was administered on day 57 of the study, and it has been shown that the effects of immunological suppression of testicular function require approximately 7 to 10 d to begin changing live performance; therefore, dietary phase 1-3 aligned with the pre-Improvest period and dietary phases 4 to 5 aligned with the post-Improvest period.

All diets were fed in the mash form and formulated with nutrient loading values for ingredients obtained from EvaPig software (version 2.0.3.2; French National Institute for Agriculture, Food and Environment Research [INRAE], METEX Animal Nutrition, and French Association of Zootechnie [AFZ]) and verified using laboratory analysis. In brief, moisture was determined with AOAC methods 935.29 and 945.15, crude protein was determined with AOAC method 990.03, crude fat was determined *via* ether extraction with AOAC method Ba 3-38, starch was determined with AOAC method 996.11, crude fiber was determined with AOAC method Ba 6a-05/ Ba 6-84, acid detergent fiber was determined with AOAC method 973.18, neutral detergent fiber was determined with AOAC method 2002.04, and ash was determined with AOAC method 942.05 (AOAC, 2016). Digestible energy (**DE**) was calculated using the equation 1-3 from the NRC (2012): DE = 4,168 − (9.1 × ash) + (1.9 × crude protein) + (3.9 × ether extract) − (3.6 × neutral detergent fiber). NE was calculated using the equation 1-8 from the NRC (2012): NE = (0.700 × DE) + (1.61× ether extract) + (0.48 × starch) − (0.91 × crude protein) − (0.87 × acid detergent fiber).

The diets for the PC males were formulated to meet 100% of the recommended ratio of SID lysine:NE for PC males for each diet phase (4.44 g SID lysine/Mcal NE for dietary phase 1, 3.68 g SID lysine/Mcal NE for dietary phase 2, 3.18 g SID lysine/Mcal NE for dietary phase 3, 2.83 g SID lysine/Mcal NE for dietary phase 4, and 2.61 g SID lysine/Mcal NE for dietary phase 5; PIC, 2021). Phase 1 diets for the Improvest males were formulated with 5.00 g SID lysine:Mcal of NE which is 12.5% greater than the recommended ratio of SID lysine:NE for PC males (PIC, 2021). Phase 2 to 5 diets for the Improvest males were formulated with 20% greater SID lysine density than recommendations for PC males (4.42 g SID lysine/Mcal NE for dietary phase 2, 3.82 g SID lysine/Mcal NE for dietary phase 3, 3.40 g SID lysine/Mcal NE for dietary phase 4, and 3.13 g SID lysine/Mcal NE for dietary phase 5).

In total, six dietary programs were fed in this study (three dietary programs for the PC males and three dietary programs for the Improvest males). Four of the dietary programs (low NE and high NE for the PC males and low NE and high NE for the Improvest males) were formulated and mixed at the mill, each of which met or exceeded additional nutrient requirements according to the NRC (2012) for PC males or Improvest males (Tables 1 and 2). In addition to the low NE and the high NE dietary programs, a 50:50 blend of the low NE and high NE diets (medium NE) was created using a robotic feeding system (FeedLogic; Wilmar, MN, USA) at the farm. The accuracy of the robotic feeding system was tested for each diet phase. This consisted of testing the accuracy with an on-farm standard operating procedure which consisted of manually weighing 15 kg samples of feed on a calibrated scale and ensuring accuracy was within 50 g (>99.5% accuracy).

**Table 1.** Composition of the low and high NE diets formulated for PC males and Improvest males during the first 3 dietary phases^1^

	Phase 1 (days 0 to 21)				Phase 2 (days 22 to 40)				Phase 3 (days 41 to 59)			
	Low NE		High NE		Low NE		High NE		Low NE		High NE	
	PC males	Improvest males	PC males	Improvest males	PC males	Improvest males	PC males	Improvest males	PC males	Improvest males	PC males	Improvest males
Ingredient, %												
Corn	—	—	40.37	36.00	—	—	48.10	43.61	—	—	58.82	51.22
Barley	42.69	41.85	—	—	49.39	48.40	—	—	59.45	58.78	—	—
Corn DDGS	20.00	20.00	20.00	20.00	20.00	20.00	20.00	20.00	20.00	20.00	20.00	20.00
Soybean meal	15.27	15.95	15.00	19.13	10.73	11.50	10.00	14.20	3.62	4.04	2.91	10.39
Fababeans	15.00	15.00	15.00	15.00	15.00	15.00	15.00	15.00	15.00	15.00	15.00	15.00
Canola meal	5.00	5.00	5.00	5.00	3.00	3.00	3.00	3.00	—	—	—	—
Canola oil	—	—	2.13	2.21	—	—	1.61	1.66	—	—	0.81	0.97
Limestone	1.27	1.27	1.28	1.24	1.20	1.19	1.21	1.19	1.15	1.15	1.15	1.13
Salt	0.46	0.46	0.45	0.45	0.46	0.46	0.45	0.45	0.46	0.46	0.45	0.45
Monocalcium phosphate	—	—	0.10	0.14	—	—	0.11	0.11	—	—	0.16	0.10
Vitamin and mineral premix^2^	0.14	0.14	0.14	0.14	0.14	0.14	0.14	0.14	0.14	0.14	0.14	0.14
l-Lysine	0.159	0.301	0.388	0.452	0.089	0.280	0.317	0.436	0.172	0.343	0.400	0.400
l-Threonine	—	0.014	0.056	0.092	—	0.018	0.033	0.099	—	0.060	0.082	0.094
dl-Methionine	—	0.022	0.058	0.107	—	0.010	0.010	0.081	—	0.012	0.029	0.065
l-Tryptophan	—	—	0.033	0.040	—	—	0.028	0.043	—	0.014	0.050	0.045
Analyzed composition, dry matter basis (unless noted)												
Dry matter, %	91.89	91.73	92.08	91.74	91.84	91.55	91.23	91.09	90.21	90.79	89.70	90.12
Crude protein, %	27.3	29.0	23.2	24.6	23.4	23.9	20.5	22.7	20.8	20.4	17.4	21.3
Ether extract, %	5.55	5.63	8.07	8.69	4.93	5.09	7.25	7.52	3.58	4.36	5.34	5.74
Starch, %	29.6	26.3	36.2	32.0	35.4	35.5	41.7	38.2	39.1	45.3	50.2	43.5
Crude fiber, %	6.51	6.66	5.28	4.91	5.31	5.08	4.58	4.91	5.05	5.21	3.96	4.17
Acid detergent fiber, %	9.15	9.84	8.05	7.74	8.85	8.48	6.75	7.90	8.41	7.88	13.20	6.71
Neutral detergent fiber, %	16.2	16.4	14.1	14.2	16.4	17.0	13.6	14.1	16.1	16.6	6.4	13.2
Ash, %	5.59	6.07	4.93	5.26	4.64	5.08	4.48	4.72	4.02	4.75	4.45	4.43
Digestible energy (DE)^3^, kcal/kg as-fed	3,502	3,482	3,653	3,655	3,483	3,430	3,597	3,603	3,390	3,355	3,651	3,531
Net energy (NE)^4^, kcal/kg as-fed	2,363	2,316	2,578	2,561	2,401	2,365	2,583	2,549	2,357	2,379	2,604	2,516
Formulation values												
Net energy (NE)^4^, kcal/kg as-fed	2,200	2,200	2,450	2,450	2,200	2,200	2,450	2,450	2,200	2,200	2,450	2,450
Standardized ileal digestible (SID) lysine, % as-fed	0.98	1.10	1.09	1.23	0.81	0.97	0.90	1.08	0.70	0.84	0.78	0.94
SID lysine:NE, g/Mcal as-fed	4.44	5.00	4.44	5.00	3.68	4.42	3.68	4.42	3.18	3.82	3.18	3.82

^1^Diets were formulated to be equivalent for SID lysine:NE for each of the dietary phases while differing in their NE.

^2^The vitamin and mineral premix provided the following per kilogram of diet: 8,000 IU vitamin A, 1,500 IU vitamin D, 30 IU vitamin E, 20 mg niacin, 12 mg D-pantothenic acid, 4 mg riboflavin, 2 mg menadione, 2 mg pyridoxine, 0.5 mg folic acid, 1 mg thiamin, 0.1 mg D-biotin, 0.02 mg vitamin B12, 100 mg Zn, 100 mg Fe, 15 mg Cu, 40 mg Mn; 1 mg I, 0.3 mg Se and 1,500 FYT Ronozyme HiPhos (DSM Nutritional Products Canada Inc., Ayr, ON, Canada).

^3^DE was calculated using equations 1 to 3 from NRC (2012): DE = 4,168 − (9.1 × ash) + (1.9 × crude protein) + (3.9 × ether extract) − (3.6 × neutral detergent fiber).

^4^NE was calculated using equations 1 to 8 from NRC (2012): NE = (0.700 × DE) + (1.61 × ether extract) + (0.48 × starch) − (0.91 × crude protein) − (0.87 × acid detergent fiber).

**Table 2.** Composition of the low and high NE diets formulated for PC males and Improvest males during the final 2 dietary phases^1^.

	Phase 4 (days 60 to 80)				Phase 5 (day 81 to marketing)			
	Low NE		High NE		Low NE		High NE	
	PC males	Improvest males	PC males	Improvest males	PC males	Improvest males	PC males	Improvest males
Ingredient, %								
Corn	—	—	67.68	60.83	—	—	68.00	63.50
Barley	68.18	67.55	—	—	75.89	75.64	5.71	3.95
Corn DDGS	15.00	15.00	15.00	15.00	10.00	10.00	10.00	10.00
Soybean meal	4.77	5.19	4.68	11.42	2.00	2.00	3.77	10.03
Fababeans	10.00	10.00	10.00	10.00	10.00	10.00	10.00	10.00
Canola oil	0.30	0.30	0.43	0.57	0.30	0.30	0.30	0.30
Limestone	1.02	1.02	1.05	1.03	1.03	1.03	1.04	1.02
Salt	0.49	0.49	0.48	0.48	0.52	0.52	0.51	0.51
Monocalcium phosphate	—	—	0.11	0.05	—	—	0.13	0.10
Vitamin and mineral premix^2^	0.10	0.10	0.10	0.10	0.10	0.10	0.10	0.10
l-Lysine	0.135	0.289	0.350	0.350	0.159	0.309	0.320	0.320
l-Threonine	—	0.048	0.071	0.083	—	0.073	0.073	0.084
dl-Methionine	—	0.01	0.017	0.050	—	0.019	0.025	0.055
l-Tryptophan	—	—	0.040	0.035	—	0.010	0.037	0.033
Analyzed composition, dry matter basis (unless noted)								
Dry matter, %	90.55	90.46	88.90	89.40	89.80	90.63	88.50	90.23
Crude protein, %	17.1	19.5	16.4	17.6	17.5	18.4	15.6	17.3
Ether extract, %	3.35	2.83	3.45	4.72	2.65	2.77	3.46	4.02
Starch, %	49.8	47.0	56.3	50.9	48.9	47.7	57.0	53.6
Crude fiber, %	4.84	5.13	3.51	4.01	4.88	5.05	3.37	3.42
Acid detergent fiber, %	7.41	7.97	4.96	5.60	6.73	7.59	5.06	5.05
Neutral detergent fiber, %	16.2	16.1	10.6	11.9	16.3	15.8	10.4	10.9
Ash, %	4.40	4.30	4.08	4.21	4.18	4.27	4.01	4.20
Digestible energy (DE)^3^, kcal/kg as-fed	3,296	3,327	3,433	3,464	3,266	3,325	3,416	3,500
Net energy (NE)^4^, kcal/kg as-fed	2,373	2,351	2,521	2,525	2,340	2,364	2,518	2,559
Formulation values								
Net energy (NE)^4^, kcal/kg as-fed	2,230	2,230	2,480	2,480	2,230	2,230	2,480	2,480
Standardized ileal digestible (SID) lysine, % as-fed	0.63	0.76	0.70	0.84	0.58	0.70	0.65	0.78
SID lysine:NE, g/Mcal as-fed	2.83	3.40	2.83	3.40	2.61	3.13	2.61	3.13

^1^Diets were formulated to be equivalent for SID lysine:NE for each of the dietary phases while differing in their NE.

^2^The vitamin and mineral premix provided the following per kilogram of diet: 8,000 IU vitamin A, 1,500 IU vitamin D, 30 IU vitamin E, 20 mg niacin, 12 mg D-pantothenic acid, 4 mg riboflavin, 2 mg menadione, 2 mg pyridoxine, 0.5 mg folic acid, 1 mg thiamin, 0.1 mg D-biotin, 0.02 mg vitamin B12, 100 mg Zn, 100 mg Fe, 15 mg Cu, 40 mg Mn; 1 mg I, 0.3 mg Se and 1,500 FYT Ronozyme HiPhos (DSM Nutritional Products Canada Inc., Ayr, ON, Canada).

^3^DE was calculated using equations 1 to 3 from NRC (2012): DE = 4,168 − (9.1 × ash) + (1.9 × crude protein) + (3.9 × ether extract) − (3.6 × neutral detergent fiber).

^4^NE was calculated using equations 1 to 8 from NRC (2012): NE = (0.700 × DE) + (1.61 × ether extract) + (0.48 × starch) − (0.91 × crude protein) − (0.87 × acid detergent fiber).

### Experimental Design

A total of six unique dietary treatment programs were used in this study, three of which were used to feed the PC males and three of which were used to feed the Improvest males. The NE levels were equal for PC males and Improvest males fed the low NE diets, PC males and Improvest males fed the medium NE diets, and PC males and Improvest males fed the high NE diets. When NE levels of the diets were expressed as a weighted average across the five dietary phases, the low NE treatment had an average NE level of 2,212 kcal/kg, the medium NE treatment had an average NE level of 2,337 kcal/kg, and the high NE treatment had an average NE level of 2,462 kcal/kg. With the NE levels consistent for PC males and Improvest males within each of the respective NE dietary treatments (SID lysine:NE levels were adjusted based on requirements for PC males and Improvest males), the experiment was treated as a 2 × 3 factorial with factors of Improvest management (PC males or Improvest males) and dietary NE level (low NE, medium NE, or high NE).

### Collection of On-Farm Data

The amount of feed delivered each day was measured using a robotic feeding system (FeedLogic). At the conclusion of each diet phase (days 21, 40, 59, 80, and 101) as well as on a weekly basis following the second dose of Improvest that did not coincide with diet phase changes (days 65, 72, 87, and 94), the amount of feed remaining was measured and used to calculate daily feed intake (*via* feed disappearance). Pen weights were collected at the start of the study, at the conclusion of each diet phase, on a weekly basis following the second dose of Improvest that did not coincide with diet phase changes, and when pigs were marketed. Gain:feed was calculated as average daily gain (**ADG**) divided by average daily feed intake (**ADFI**). Caloric intake and lysine intake were the product of formulated dietary composition and ADFI. Caloric intake per unit of gain (caloric intake:gain) and lysine intake per unit of gain (lysine intake:gain) were calculated as caloric intake divided by ADG and lysine intake divided by ADG, respectively.

It should be noted that marketing events began on day 83, thus growth performance calculations on day 83 and, thereafter, were reflective of the pigs remaining in the pen. Weights of the marketed pigs were collected on each of the marketing days. The number of pigs dead or removed from each pen throughout the study was recorded and growth performance calculations thereafter were reflective of the pigs remaining in the pen.

### Marketing and Slaughter Procedures

An equal number of pigs were marketed from each of the six treatment groups on day 83 (21.0% of the population), day 90 (13.0% of the population), day 97 (44.3% of the population), and day 101 (21.7% of the population) of the experiment. These days coincided with 26, 33, 40, and 44 d post-second Improvest dose. Thus, the weighted average for time post-second Improvest dose to marketing was 33.0 d.

Pigs were slaughtered under commercial conditions and identification was maintained throughout the slaughter process with a tattoo that reflected the pen number. Hot carcass weights (HCW) were recorded by processing plant personnel and carcass dressing percentage was calculated as HCW divided by weight at marketing. Grading probe measurements (i.e., backfat thickness and muscle depth) were collected online using the left side of carcasses by experienced operators from provincial grading authorities using a Destron PG-100 optical grading probe (International Destron Technologies). The grading probe was inserted perpendicularly at the grading site between the third and fourth last ribs, 7 cm off the split line according to Canadian grading standards (Pomar and M. Marcoux, 2003). Backfat thickness and muscle depth (i.e., loin depth) measurements were used to obtain the predicted lean yield value for each carcass using the following equation (Bohrer et al., 2023):


$$\begin{array}{l} \begin{array}{llllll} & Destron\;predicted\;lean\;yield\;\left(2023\;equation\right)\; & \;=\;89.16298\;--(1.63023\times backfat\;thickness)--(0.42126\; & \;\times muscle\;depth+(0.01930\times backfat\;thickness^{2})\; & \;+(0.00308\times muscle\;depth^{2})+(0.00369\times backfat\; & \;thickness\times muscle\;depth).\; \end{array} \end{array}$$

where backfat thickness (mm) and muscle depth (mm) are collected at the grading site for each carcass.

### Statistical Analysis

There was a total of eight pen replicates for each treatment interaction (Improvest management × dietary NE treatment). Pen was considered as the experimental unit for all analyses. Live weights were analyzed with PROC MIXED of SAS (v. 9.4, SAS Inst. Inc.; Cary, NC, USA) as repeated measures over time with fixed effects of Improvest management, dietary treatment, and their interaction and a random effect of replication. An autoregressive covariance structure was selected based on the analysis of the fit statistics. A slice statement was used to detect statistical differences at each time. All other parameters were analyzed with PROC MIXED of SAS with fixed effects of Improvest management, dietary treatment, and their interaction and a random effect of replication. Differences between Improvest management, dietary treatment, and their interaction were considered significant at *P* ≤ 0.05.

## Results

### Live Weights

#### Pre-Improvest period

There were no significant interactions between Improvest management and NE (*P* ≥ 0.22) for the live weight of pigs during the pre-Improvest period (the second dose of Improvest was administered on day 57) (Table 3). There was a difference in live weight for the main effect of Improvest management on day 40 of the study where Improvest males were 2.4 kg lighter (*P* = 0.01) compared with PC males. This was the only point in the study where PC males were significantly heavier than Improvest males; however, PC males were numerically heavier than Improvest males on day 0 of the study and throughout the pre-Improvest period. There were no significant differences (*P* ≥ 0.33) in the live weight of pigs during the pre-Improvest period attributed to the main effect of NE.

**Table 3.** Effects of dietary NE on live weights for male market pigs managed with physical castration or with Improvest^1^^,^^2^

	Improvest management			NE level				*P* values		
	PC males	Improvest males	SEM	Low NE	Medium NE	High NE	SEM	Improvest	NE	Interaction
Weights^3^^,^^4^										
Starting weight (day 0), kg	28.7	27.8	0.6	28.3	28.2	28.2	0.6	0.35	0.99	0.97
Day 21, kg	51.0	49.4	0.6	49.8	50.49	50.3	0.6	0.10	0.85	0.69
Day 40, kg	73.1^a^	70.7^b^	0.8	71.4	72.2	72.0	0.7	0.01	0.78	0.22
Day 59, kg	95.4	93.8	0.9	93.7	95.4	94.7	1.0	0.11	0.33	0.41
Day 65, kg	101.3	99.5	0.9	99.8	101.1	100.2	1.0	0.07	0.50	0.39
Day 72, kg	109.7	109.4	0.9	108.4	110.6	109.7	1.0	0.73	0.17	0.42
Day 80, kg	119.2^b^	121.2^a^	0.9	118.9	121.4	120.4	0.9	0.04	0.10	0.11
Day 87, kg	124.3^b^	128.4^a^	0.6	125.5	127.5	125.9	0.7	<0.01	0.20	<0.01
Day 94, kg	131.2^b^	135.6^a^	0.9	132.3	134.1	133.8	1.0	<0.01	0.28	<0.01
Day 101, kg	128.7^b^	133.8^a^	1.6	128.5^y^	131.9^xy^	133.3^x^	1.8	<0.01	<0.01	<0.01
Live weight at marketing, kg	136.8^b^	141.0^a^	0.8	137.9	139.5	139.3	0.8	<0.01	0.21	<0.01
Days to marketing following last full pen weight^5^, days	15.0	15.0	0.6	15.0	15.0	15.0	0.6	0.84	0.99	0.33

^a,b,x,y^Least squares means within each row of main effects with different superscripts are significantly different (*P* < 0.05).

^1^Improvest is a gonadotropin-releasing factor (GnRF) analog-diphtheria toxoid conjugate product approved for temporary suppression of testicular function in intact male pigs (Zoetis Canada Inc.); Improvest pigs received the first dose of Improvest on day 29 of the study and the second dose of Improvest on day 57 of the study; PC males = physically castrated males.

^2^Within each diet phase, diets were formulated to meet SID lysine:NE requirements for PC males or Improvest males while differing in their NE; medium NE diets were a 50:50 blend of the low NE and high NE diets mixed at the farm; low NE treatment had an average NE level of 2,212 kcal/kg, medium NE treatment had an average NE level of 2,337 kcal/kg, high NE treatment had an average NE level of 2,462 kcal/kg.

^3^Pigs were approximately 10 wk old at the beginning of the study.

^4^An equal number of pigs were marketed from each of the six treatment groups on day 83 (21.0% of the population), day 90 (13.0% of the population), day 97 (44.3% of the population), and day 101 (21.7% of the population) of the study, thus weights on day 87, 94, and 101 were reflective of the pigs remaining in the pen on those respective days.

^5^The average number of days from the last full pen weight (day 80) to the day when all pigs were marketed.

#### Post-Improvest period

**Figure 2.**
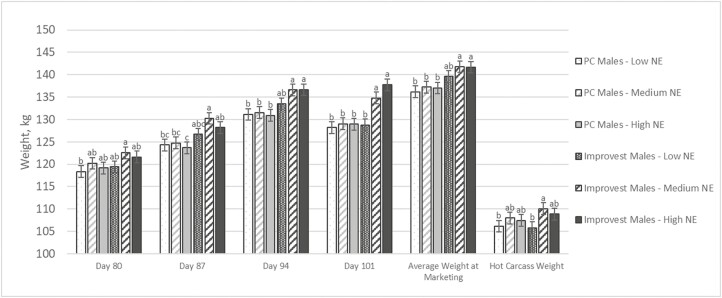
Interactive effects of dietary NE and Improvest management on live weights; least squares means within each day with different letters are significantly different (*P* < 0.05); Improvest is a gonadotropin-releasing factor (GnRF) analog-diphtheria toxoid conjugate product approved for temporary suppression of testicular function in intact male pigs (Zoetis Canada Inc.); Improvest pigs received the first dose of Improvest on day 29 of the study and the second dose of Improvest on day 57 of the study; PC males = physically castrated males; Within each diet phase, diets were formulated to meet SID lysine:NE requirements for PC males or Improvest males while differing in their NE; medium NE diets were a 50:50 blend of the low NE and high NE diets mixed at the farm; low NE treatment had an average NE level of 2,212 kcal/kg, medium NE treatment had an average NE level of 2,337 kcal/kg, high NE treatment had an average NE level of 2,462 kcal/kg; an equal number of pigs were marketed from each of the six treatment groups on day 83 (21.0% of the population), day 90 (13.0% of the population), day 97 (44.3% of the population), and day 101 (21.7% of the population) of the study, thus weights on days 87, 94, and 101 were reflective of the pigs remaining in the pen on those respective days.

There were no significant interactions (*P* > 0.05) between Improvest management and NE for live weight until day 87 of the study (Table 3; Figure 2). Prior to the interpretation of the study results, it should be mentioned that marketing began on day 83 of the study. Therefore, weights on day 87 and thereafter were partial pen weights with the fastest-growing pigs already marketed. The interactions between Improvest management and NE were attributed to differing levels of impact for NE between the Improvest males and the PC males. For instance, Improvest males had numerically heavier (*P* = 0.14 to 0.17) live weights at marketing when fed the medium NE and high NE diets compared with the low NE diets (Improvest males fed low NE = 139.6 kg; Improvest males fed medium NE = 141.8 kg; Improvest males fed high NE = 141.7 kg) while a smaller numerical difference (*P* = 0.98 to 0.99) in live weight at marketing was detected for the different NE treatments among PC males (PC males fed low NE = 136.2 kg; PC males fed medium NE = 137.2 kg; PC males fed high NE = 137.0 kg). This resulted in the magnitude of change for live weight at marketing between Improvest males and PC males of 3.4 kg when pigs were fed low NE (*P* = 0.19), 4.6 kg when pigs were fed medium NE (*P* = 0.04), and 4.7 kg when pigs were fed high NE (*P* = 0.03).

### Live Performance Calculations

Live performance parameters were reported for the pre-Improvest period (days 0 to 59), post-Improvest period (day 60 to marketing), and overall period (day 0 to marketing) (Table 4); as well as for intervals between 0 to 1, 1 to 2, 2 to 3, 3 to 4, 4 to 5, and 5 to 6 wk following the second dose of Improvest (Table 5).

**Table 4.** Effects of dietary NE on live performance (ADFI, ADG, and G:F) for male market pigs managed with physical castration or with Improvest^1^^,^^2^

	Improvest management			NE level				*P* values		
	PC males	Improvest males	SEM	Low NE	Medium NE	High NE	SEM	Improvest	NE	Interaction
Pre-Improvest period (day 0 to 59)										
Average daily feed intake, g/d	2,658^a^	2,313^b^	34	2,588^x^	2,480^y^	2,389^z^	37	<0.01	<0.01	0.71
Average daily gain, g/d	1,121	1,116	8	1,103	1,131	1,122	9	0.62	0.06	0.74
Gain:Feed	0.423^b^	0.484^a^	0.004	0.429^z^	0.459^y^	0.472^x^	0.005	<0.01	<0.01	0.63
Post-Improvest period (days 60 to marketing)										
Average daily feed intake, g/d	3,791^b^	3,960^a^	28	3,980^x^	3,926^x^	3,720^y^	34	<0.01	<0.01	0.53
Average daily gain, g/d	1,135^b^	1,303^a^	11	1,209	1,216	1,232	12	<0.01	0.30	0.32
Gain:feed	0.300^b^	0.330^a^	0.003	0.304^y^	0.310^y^	0.331^x^	0.003	<0.01	<0.01	0.20
Overall period (days 0 to marketing)										
Average daily feed intake, g/d	3,071^a^	2,908^b^	27	3,089^x^	3,006^x^	2,873^y^	30	<0.01	<0.01	0.76
Average daily gain, g/d	1,126^b^	1,183^a^	6	1,141^y^	1,160^x^	1,161^x^	7	<0.01	0.02	0.19
Gain:feed	0.367^b^	0.408^a^	0.003	0.370^z^	0.387^y^	0.405^x^	0.003	<0.01	<0.01	0.33

^a,b,x,y,z^Least squares means within each row of main effects with different superscripts are significantly different (*P* < 0.05).

^1^Improvest is a gonadotropin-releasing factor (GnRF) analog-diphtheria toxoid conjugate product approved for temporary suppression of testicular function in intact male pigs (Zoetis Canada Inc.); Improvest pigs received the first dose of Improvest on day 29 of the study and the second dose of Improvest on day 57 of the study; PC males = physically castrated males.

^2^Within each diet phase, diets were formulated to meet SID lysine:NE requirements for PC males or Improvest males while differing in their NE; medium NE diets were a 50:50 blend of the low NE and high NE diets mixed at the farm; low NE treatment had an average NE level of 2,212 kcal/kg, medium NE treatment had an average NE level of 2,337 kcal/kg, high NE treatment had an average NE level of 2,462 kcal/kg.

**Table 5.** Interactive effects of dietary NE and Improvest management on live performance (ADFI, ADG, and G:F) during the post-second dose period^1^^,^^2^^,^^3^

	Treatment							*P* values		
	PC males—low NE	PC males—medium NE	PC males—high NE	Improvest males—low NE	Improvest males—medium NE	Improvest males—high NE	SEM	Improvest	NE	Interaction
0 to 1 wk post-second dose (days 60 to 65)										
Average daily feed intake, g/d	3,616^a^	3,398^ab^	3,317^ab^	3,125^bc^	2,980^c^	2,836^c^	76	<0.01	<0.01	0.87
Average daily gain, g/d	1,044	963	927	939	938	897	44	0.45	0.21	0.61
Gain:Feed	0.289	0.285	0.279	0.300	0.315	0.315	0.012	0.01	0.90	0.54
1 to 2 wk post-second dose (days 66 to 72)										
Average daily feed intake, g/d	3,748^a^	3,686^ab^	3,401^bc^	3,185^c^	3,668^ab^	3,496^abc^	84	0.02	0.01	<0.01
Average daily gain, g/d	1,179^c^	1,183^c^	1,250^bc^	1,263^bc^	1,474^a^	1,421^ab^	41	<0.01	0.01	0.05
Gain:feed	0.315^c^	0.322^bc^	0.367^ab^	0.400^a^	0.402^a^	0.407^a^	0.012	<0.01	0.04	0.13
2 to 3 wk post-second dose (days 73 to 80)										
Average daily feed intake, g/d	4,117^ab^	3,826^bc^	3,663^c^	4,253^a^	4,052^ab^	4,070^ab^	72	<0.01	<0.01	0.18
Average daily gain, g/d	1,116^b^	1,217^b^	1,195^b^	1,488^a^	1,438^a^	1,477^a^	38	<0.01	0.65	0.15
Gain:Feed	0.271^c^	0.318^b^	0.327^ab^	0.351^ab^	0.355^ab^	0.363^a^	0.009	<0.01	<0.01	0.03
3 to 4 wk post-second dose (days 81 to 87)										
Average daily feed intake, g/d	3,856^b^	3,987^b^	3,814^b^	4,852^a^	4,624^a^	3,736^b^	72	<0.01	<0.01	<0.01
Average daily gain, g/d	1,160^b^	1,087^b^	1,078^b^	1,470^a^	1,502^a^	1,403^a^	67	<0.01	0.32	0.54
Gain:Feed	0.302^b^	0.272^b^	0.283^b^	0.303^b^	0.325^ab^	0.375^a^	0.016	<0.01	0.07	0.01
4 to 5 wk post-second dose (days 88 to 94)										
Average daily feed intake, g/d	4,135^b^	4,332^b^	4,297^b^	4,866^a^	4,965^a^	4,766^a^	103	<0.01	0.19	0.30
Average daily gain, g/d	1,217	1,224	1,312	1,250	1,204	1,466	77	0.32	0.03	0.42
Gain:feed	0.294	0.284	0.305	0.258	0.244	0.310	0.018	0.07	0.02	0.30
5 to 6 wk post-second dose (days 95 to 101)										
Average daily feed intake, g/d	4,103^abc^	3,727^bc^	3,530^c^	4,750^a^	4,443^a^	4,239^ab^	173	<0.01	<0.01	0.97
Average daily gain, g/d	1,162	1,010	1,016	1,167	1,069	1,145	120	0.47	0.51	0.85
Gain:feed	0.282	0.271	0.291	0.238	0.238	0.268	0.025	0.09	0.53	0.91

^a,b,c^Least squares means within each row of interactive effects with different superscripts are significantly different (*P* < 0.05).

^1^An equal number of pigs were marketed from each of the six treatment groups on day 83 (21.0% of the population), day 90 (13.0% of the population), day 97 (44.3% of the population), and day 101 (21.7% of the population) of the study, thus weights on days 87, 94, and 101 were reflective of the pigs remaining in the pen on those respective days.

^2^Improvest is a gonadotropin-releasing factor (GnRF) analog-diphtheria toxoid conjugate product approved for temporary suppression of testicular function in intact male pigs (Zoetis Canada Inc.); Improvest pigs received the first dose of Improvest on day 29 of the study and the second dose of Improvest on day 57 of the study; PC males = physically castrated males.

^3^Within each diet phase, diets were formulated to meet SID lysine:NE requirements for PC males or Improvest males while differing in their NE; medium NE diets were a 50:50 blend of the low NE and high NE diets mixed at the farm; low NE treatment had an average NE level of 2,212 kcal/kg, medium NE treatment had an average NE level of 2,337 kcal/kg, high NE treatment had an average NE level of 2,462 kcal/kg.

#### Pre-Improvest period

There were no significant interactions between Improvest management and NE (*P* ≥ 0.63) for ADFI, ADG, or feed efficiency during the pre-Improvest period (Table 4). There was a difference in ADFI and feed efficiency between Improvest males and PC males. Improvest males consumed less feed (13.0% lower ADFI; *P* < 0.01) and grew at the same rate (ADG: *P* = 0.62), which resulted in 14.4% greater G:F (*P* < 0.01) compared with PC males during the pre-Improvest period. There were differences in ADFI attributed to NE during the pre-Improvest period, yet there were no differences (*P *= 0.06) in ADG attributed to NE during the pre-Improvest period. Pigs fed low NE diets consumed more feed compared with the other two treatments (4.4% greater ADFI than medium NE and 8.3% greater ADFI than high NE; *P* < 0.01) and pigs fed medium NE diets consumed more feed compared to pigs fed high NE (3.8% greater ADFI; *P* < 0.01) during the pre-Improvest period. This resulted in lower levels of feed efficiency for pigs fed low NE diets compared with the other two treatments (6.5% lower G:F than medium NE and 9.1% lower than high NE; *P* < 0.01) during the pre-Improvest period. In addition, pigs fed medium NE diets had lower levels of feed efficiency compared to pigs fed high NE (2.8% lower G:F; *P* < 0.01) during the pre-Improvest period.

#### Post-Improvest period

There were no significant interactions between Improvest management and NE (*P* ≥ 0.20) for ADFI, ADG, or feed efficiency during the post-Improvest period (Table 4). There were significant main effects for Improvest management and NE during the post-Improvest period. Improvest males consumed more feed (4.5% greater ADFI; *P* < 0.01), grew faster (14.8% greater ADG; *P* < 0.01), and were more efficient (10.0% greater G:F; *P* < 0.01) compared with PC males during the post-Improvest period. Pigs fed low NE and medium NE diets consumed more feed (7.0% greater ADFI for low NE and 5.5% greater ADFI for medium NE; *P* < 0.01) and grew at the same rate (ADG: *P* = 0.30) compared with pigs fed high NE, which resulted in lower levels of feed efficiency (8.2% lower G:F for low NE and 6.3% lower G:F for medium NE; *P* < 0.01) compared with pigs fed high NE during the post-Improvest period.

To further elucidate differences in ADFI, ADG, and feed efficiency, calculations for each weekly interval following the second dose of Improvest were evaluated. The interaction between Improvest management and NE was significant in several instances for each of the parameters of interest (Table 5). Such instances included the interaction between Improvest management and NE for ADFI and ADG during the time period of 1 to 2 wk post-second dose (PC males fed low NE and medium NE diets consumed more feed and grew slower than PC males fed high NE diets while Improvest males fed low NE diets consumed less feed and grew slower than Improvest males fed medium NE and high NE diets), the interaction between Improvest management and NE for G:F during the time period of 2 to 3 wk post-second dose (PC males fed low NE diets were less efficient compared with PC males fed high NE diets while Improvest males had similar levels of efficiency across the NE treatments), and the interaction between Improvest management and NE for ADFI and G:F during the time period of 3 to 4 wk post-second dose (PC males consumed similar levels of feed and had similar G:F across the NE treatments while Improvest males consumed more feed and were less efficient when fed low NE diets compared with high NE diets).

ADFI was significantly affected for each weekly time interval during the post-second dose period for the main effect of Improvest management. Improvest males consumed 13.5% less feed 0 to 1 wk post-second dose, 4.5% less feed 1 to 2 wk post-second dose, 6.6% more feed 2 to 3 wk post-second dose, 13.3% more feed 3 to 4 wk post-second dose, 14.4% more feed 4 to 5 wk post-second dose, and 18.2% more feed 5 to 6 wk post-second dose compared with PC males. For the main effect of Improvest management, ADG was not significantly affected from 0 to 1, 4 to 5, and 5 to 6 wk post-second dose, but was significantly affected from 1 to 2, 2 to 3, and 3 to 4 wk post-second dose. Improvest males grew 15.1% faster 1 to 2 wk post-second dose, 24.8% faster 2 to 3 wk post-second dose, and 31.6% faster 3 to 4 wk post-second dose compared with PC males. For the main effect of Improvest management, G:F was significantly affected from 0 to 1, 1 to 2, 2 to 3, and 3 to 4 wk post-second dose and was not significantly affected from 4 to 5 and 5 to 6 wk post-second dose. Improvest males were 9.2% more efficient 0 to 1 wk post-second dose, 20.3% more efficient 1 to 2 wk post-second dose, 16.7% more efficient 2 to 3 wk post-second dose, and 16.8% more efficient 3 to 4 wk post-second dose compared with PC males. While not significant (but approaching significant levels; *P* < 0.10), Improvest males had lower numerical values for G:F compared with PC males from 4 to 5 wk post-second dose (difference of 7.8%) and 5 to 6 wk post-second dose (difference of 11.7%).

For the weekly time intervals, there were several instances where NE significantly affected ADFI, ADG, and feed efficiency. In most instances, pigs fed low NE diets consumed more feed and grew at similar or slower rates than pigs fed high NE diets, with pigs fed medium NE diets having intermediate values. These differences can be largely explained by results discussed for the post-Improvest period.

#### Overall period

There were no significant interactions between Improvest management and NE (*P* ≥ 0.19) for ADFI, ADG, or feed efficiency during the entire grow-finish period (day 0 to marketing; Table 4). There were significant main effects for Improvest management and NE for the overall period. Improvest males consumed less feed (5.3% lower ADFI; *P* < 0.01), grew faster (5.1% greater ADG; *P* < 0.01), and were more efficient (11.2% greater G:F; *P* < 0.01) compared with PC males for the overall period. Pigs fed low NE and medium NE diets consumed more feed (7.5% greater ADFI for low NE and 4.6% greater ADFI for medium NE; *P* < 0.01) and pigs fed low NE diets grew slower compared with the other two treatments (1.6% lower ADG compared with medium NE and 1.7% lower ADG compared with high NE; *P* < 0.01) for the overall period. This resulted in pigs fed low NE diets having the lowest levels of feed efficiency (4.4% lower G:F than medium NE and 8.6% lower G:F than high NE; *P* < 0.01) for the overall period. In addition, pigs fed medium NE diets were less feed efficient (4.4% lower G:F; *P* < 0.01) compared with pigs fed high NE diets for the overall period.

### Caloric and Lysine Intake and Efficiency

Caloric intake, caloric intake:gain, lysine intake, and lysine intake:gain were reported for the pre-Improvest period (days 0 to 59), post-Improvest period (day 60 to marketing), and overall period (day 0 to marketing) (Table 6); as well as for intervals between 0 to 1, 1 to 2, 2 to 3, 3 to 4, 4 to 5, and 5 to 6 wk following the second dose of Improvest (Table 7). Caloric intake:gain and lysine intake:gain were calculated as caloric intake divided by ADG and lysine intake divided by ADG, respectively. Therefore, greater values in caloric intake:gain and lysine intake:gain should be interpreted as a greater number of calories (kcal) or lysine (g SID) consumed to achieve a kg of ADG.

**Table 6.** Effects of dietary NE on caloric and lysine intake and efficiency for male market pigs managed with physical castration or with Improvest^1^^,^^2^^,^^3^

	Improvest management			NE level				*P* values		
	PC males	Improvest males	SEM	Low NE	Medium NE	High NE	SEM	Improvest	NE	Interaction
Pre-Improvest period (days 0 to 59)										
Caloric intake, kcal NE/d	6,187^a^	5,378^b^	79	5,707	5,777	5,864	86	<0.01	0.20	0.89
Caloric intake:gain, kcal NE/kg gain	5,485^a^	4,779^b^	50	5,136	5,068	5,192	56	<0.01	0.15	0.89
Lysine intake, g SID lysine/d	22.76^b^	23.36^a^	0.26	22.74	23.02	23.43	0.34	0.03	0.11	0.88
Lysine intake:gain, g SID lysine/kg gain	20.28^b^	20.91^a^	0.20	20.62	20.31	20.86	0.22	<0.01	0.08	0.86
Post-Improvest period (days 60 to marketing)										
Caloric intake, kcal NE/d	9,001^b^	9,574^a^	71	9,055^y^	9,430^x^	9,378^x^	86	<0.01	0.01	0.42
Caloric intake:gain, kcal NE/kg gain	7,934^a^	7,325^b^	70	7,475^y^	7,800^x^	7,614^xy^	83	<0.01	0.02	0.09
Lysine intake, g SID lysine/d	24.42^b^	31.15^a^	0.21	27.09^y^	28.22^x^	28.05^x^	0.25	<0.01	0.01	0.40
Lysine intake:gain, g SID lysine/kg gain	21.53^b^	23.85^a^	0.20	22.26^y^	23.18^x^	22.63^xy^	0.24	<0.01	0.02	0.09
Overall period (days 0 to marketing)										
Caloric intake, kcal NE/d	7,357^a^	7,123^b^	68	7,099^y^	7,296^xy^	7,325^x^	77	<0.01	0.03	0.88
Caloric intake:gain, kcal NE/kg gain	6,503^a^	5,838^b^	49	6,108	6,204	6,199	56	<0.01	0.30	0.27
Lysine intake, g SID lysine/d	23.45^b^	26.60^a^	0.24	24.55^y^	25.18^xy^	25.35^x^	0.27	<0.01	0.02	0.90
Lysine intake:gain, g SID lysine/kg gain	20.80^b^	22.14^a^	0.16	21.30	21.51	21.60	0.19	<0.01	0.38	0.34

^a,b,x,y^Least squares means within each row of main effects with different superscripts are significantly different (*P* < 0.05).

^1^Caloric intake and lysine intake were the product of formulated dietary composition and ADFI.

^2^Improvest is a gonadotropin-releasing factor (GnRF) analog-diphtheria toxoid conjugate product approved for temporary suppression of testicular function in intact male pigs (Zoetis Canada Inc.); Improvest pigs received the first dose of Improvest on day 29 of the study and the second dose of Improvest on day 57 of the study; PC males = physically castrated males.

^3^Within each diet phase, diets were formulated to meet SID lysine:NE requirements for PC males or Improvest males while differing in their NE; medium NE diets were a 50:50 blend of the low NE and high NE diets mixed at the farm; low NE treatment had an average NE level of 2,212 kcal/kg, medium NE treatment had an average NE level of 2,337 kcal/kg, high NE treatment had an average NE level of 2,462 kcal/kg.

**Table 7.** Interactive effects of dietary NE and Improvest management on caloric and lysine intake and efficiency during the post-second dose period^1^^,^^2^^,^^3^^,^^4^

	Treatment							*P* values		
	PC males—low NE	PC males—medium NE	PC males—high NE	Improvest males—low NE	Improvest males—medium NE	Improvest males—high NE	SEM	Improvest	NE	Interaction
0 to 1 wk post-second dose (days 60 to 65)										
Caloric intake, kcal NE/d	8,063^a^	8,003^a^	8,226^a^	6,968^b^	7,018^b^	7,034^b^	176	<0.01	0.73	0.83
Caloric intake:gain, kcal NE/kg gain	7,880^ab^	8,333^ab^	8,925^a^	7,542^b^	7,515^b^	7,981^ab^	311	0.01	0.06	0.58
Lysine intake, g SID lysine/d	22.78	22.60	23.22	23.75	23.84	23.83	0.54	0.04	0.82	0.83
Lysine intake:gain, g SID lysine/kg gain	22.26^b^	23.53^ab^	25.19^ab^	25.71^ab^	25.53^ab^	27.03^a^	0.97	<0.01	0.07	0.64
1 to 2 wk post-second dose (days 66 to 72)										
Caloric intake, kcal NE/d	8,357^a^	8,680^a^	8,435^a^	7,102^b^	8,638^a^	8,671^a^	194	0.03	<0.01	<0.01
Caloric intake:gain, kcal NE/kg gain	7,116^a^	7,361^a^	6,781^ab^	5,675^c^	5,873^c^	6,134^bc^	193	<0.01	0.51	0.06
Lysine intake, g SID lysine/d	23.61^b^	24.51^b^	23.81^b^	24.20^b^	29.35^a^	29.37^a^	0.61	<0.01	<0.01	<0.01
Lysine intake:gain, g SID lysine/kg gain	20.10	20.78	19.14	19.34	19.95	20.78	0.61	0.98	0.57	0.08
2 to 3 wk post-second dose (days 73 to 80)										
Caloric intake, kcal NE/d	9,180^b^	9,010^b^	9,085^b^	9,485^ab^	9,542^ab^	10,093^a^	171	<0.01	0.16	0.13
Caloric intake:gain, kcal NE/kg gain	8,251^a^	7,413^bc^	7,630^ab^	6,442^d^	6,646^cd^	6,866^bcd^	188	<0.01	0.24	0.01
Lysine intake, g SID lysine/d	25.94^b^	25.44^b^	25.64^b^	32.33^a^	32.42^a^	34.18^a^	0.53	<0.01	0.16	0.13
Lysine intake:gain, g SID lysine/kg gain	23.31	20.93	21.54	21.96	22.58	23.26	0.60	0.18	0.33	0.02
3 to 4 wk post-second dose (days 81 to 87)										
Caloric intake, kcal NE/d	8,599^c^	9,390^b^	9,459^b^	10,819^a^	10,890^a^	9,266^bc^	172	<0.01	<0.01	<0.01
Caloric intake:gain, kcal NE/kg gain	7,637^abc^	8,938^ab^	9,017^a^	7,418^bc^	7,282^c^	6,741^c^	466	<0.01	0.29	0.02
Lysine intake, g SID lysine/d	22.37^d^	24.52^c^	24.79^c^	33.96^a^	34.22^a^	29.14^b^	0.49	<0.01	<0.01	<0.01
Lysine intake:gain, g SID lysine/kg gain	19.86	23.34	23.63	23.29	22.88	21.20	1.26	0.83	0.31	0.02
4 to 5 wk post-second dose (days 88 to 94)										
Caloric intake, kcal NE/d	9,221^d^	10,202^c^	10,658^c^	10,852^bc^	11,692^ab^	11,821^a^	246	<0.01	<0.01	0.49
Caloric intake:gain, kcal NE/kg gain	7,683^b^	8,575^ab^	8,516^ab^	8,751^ab^	9,883^a^	8,316^ab^	547	0.08	0.11	0.28
Lysine intake, g SID lysine/d	23.98^d^	26.64^c^	27.93^c^	34.06^b^	36.74^a^	37.18^a^	0.74	<0.01	<0.01	0.73
Lysine intake:gain, g SID lysine/kg gain	19.98^c^	22.39^bc^	22.32^bc^	27.47^ab^	31.06^a^	26.15^abc^	1.59	<0.01	0.10	0.24
5 to 6 wk post-second dose (days 95 to 101)										
Caloric intake, kcal NE/d	9,151^b^	8,776^b^	8,754^b^	10,591^a^	10,463^a^	10,513^a^	406	<0.01	0.73	0.90
Caloric intake:gain, kcal NE/kg gain	8,261	9,067	8,961	10,500	11,081	9,837	1,066	0.05	0.74	0.77
Lysine intake, g SID lysine/d	23.80^b^	22.92^b^	22.94^b^	33.25^a^	32.88^a^	33.06^a^	1.23	<0.01	0.83	0.95
Lysine intake:gain, g SID lysine/kg gain	21.49^b^	23.68^ab^	23.49^ab^	32.96^ab^	34.82^a^	30.94^ab^	3.22	<0.01	0.75	0.77

^a,b,c,d^Least squares means within each row of interactive effects with different superscripts are significantly different (*P* < 0.05).

^1^Caloric intake and lysine intake were the product of formulated dietary composition and ADFI.

^2^An equal number of pigs were marketed from each of the six treatment groups on day 83 (21.0% of the population), day 90 (13.0% of the population), day 97 (44.3% of the population), and day 101 (21.7% of the population) of the study, thus weights on days 87, 94, and 101 were reflective of the pigs remaining in the pen on those respective days.

^3^Improvest is a gonadotropin-releasing factor (GnRF) analog-diphtheria toxoid conjugate product approved for temporary suppression of testicular function in intact male pigs (Zoetis Canada Inc.); Improvest pigs received the first dose of Improvest on day 29 of the study and the second dose of Improvest on day 57 of the study; PC males = physically castrated males.

^4^Within each diet phase, diets were formulated to meet SID lysine:NE requirements for PC males or Improvest males while differing in their NE; medium NE diets were a 50:50 blend of the low NE and high NE diets mixed at the farm; low NE treatment had an average NE level of 2,212 kcal/kg, medium NE treatment had an average NE level of 2,337 kcal/kg, high NE treatment had an average NE level of 2,462 kcal/kg.

#### Pre-Improvest period

There were no significant interactions between Improvest management and NE (*P* ≥ 0.86) in caloric intake, caloric intake:gain, lysine intake, or lysine intake:gain during the pre-Improvest period (Table 6). There were significant differences in caloric intake, caloric intake:gain, lysine intake, and lysine intake:gain between Improvest males and PC males during the pre-Improvest period. Improvest males had lower caloric intake (13.1% lower; *P* < 0.01), lower levels of caloric intake:gain (12.9% lower; *P* < 0.01), greater lysine intake (2.6% greater; *P* = 0.03), and greater levels of lysine intake:gain (3.1% greater; *P* < 0.01) compared with PC males during the pre-Improvest period. There were no significant differences (*P* ≥ 0.08) in caloric intake, caloric intake:gain, lysine intake, or lysine intake:gain for the main effect of NE during the pre-Improvest period.

#### Post-Improvest period

There were no significant interactions between Improvest management and NE (*P* ≥ 0.09) in caloric intake, caloric intake:gain, lysine intake, or lysine intake:gain during the post-Improvest period (Table 6). There were significant differences in caloric intake, caloric intake:gain, lysine intake, and lysine intake:gain between Improvest males and PC males during the post-Improvest period. Improvest males had greater caloric intake (5.3% greater; *P* < 0.01), lower levels of caloric intake:gain (7.7% lower; *P* < 0.01), greater lysine intake (27.6% greater; *P* = 0.03), and greater levels of lysine intake:gain (10.8% greater; *P* < 0.01) compared with PC males during the post-Improvest period. There were significant differences in caloric intake, caloric intake:gain, lysine intake, and lysine intake:gain for the main effect of NE during the post-Improvest period. Pigs fed low NE diets had lower caloric intake and lysine intake compared with pigs fed the other two NE treatments (4.0% lower compared with medium NE diets and 3.4% lower compared with high NE diets; *P* = 0.01) during the post-Improvest period. Pigs fed low NE diets had lower levels of caloric intake:gain and lysine intake:gain compared with pigs fed medium NE (4.2% lower in caloric intake:gain and 4.0% lower for lysine intake:gain; *P* < 0.02) while pigs fed high NE diets were intermediate and not different from the other treatments during the post-Improvest period.

The interaction between Improvest management and NE was significant in several instances in caloric intake, caloric intake:gain, lysine intake, and lysine intake:gain for each weekly interval following the second dose of Improvest (Table 7). During the time period of 1 to 2 wk post-second dose, Improvest males fed low NE diets had lower levels of caloric intake and lysine intake compared to Improvest males fed medium and high NE diets, while caloric intake and lysine intake levels were similar for PC males across the NE treatments. During the time period of 2 to 3 wk post-second dose, Improvest males fed low NE diets had slightly lower levels of caloric intake:gain and lysine intake:gain compared with Improvest males fed medium NE and high NE diets while PC males fed low NE diets had greater levels of caloric intake:gain and lysine intake:gain compared with PC males fed medium NE diets. During the time period of 3 to 4 wk post-second dose, Improvest males fed low NE and medium NE diets had greater levels of caloric intake, caloric intake:gain, lysine intake, and lysine intake:gain with Improvest males fed high NE diets while PC males fed low NE diets had lower levels of caloric intake, caloric intake:gain, lysine intake, and lysine intake:gain compared with PC males fed high NE diets.

Caloric intake and lysine intake were significantly affected for each weekly time interval during the post-second dose period for the main effect of Improvest management (Table 7). Improvest males consumed 13.5% less kcal NE and 4.1% more SID lysine 0 to 1 wk post-second dose, 4.2% less kcal NE and 15.3% more SID lysine 1 to 2 wk post-second dose, 6.8% more kcal NE and 28.5% more SID lysine 2 to 3 wk post-second dose, 12.9% more kcal NE and 35.8% more SID lysine 3 to 4 wk post-second dose, 14.2% more kcal NE and 37.4% more SID lysine 4 to 5 wk post-second dose, and 18.3% more kcal NE and 42.4% more SID lysine 5 to 6 wk post-second dose. Caloric intake:gain was significantly affected from 0 to 1, 1 to 2 , 2 to 3, 3 to 4, and 5 to 6 wk post-second dose and was not significantly affected from 4 to 5 wk post-second dose. Improvest males had 8.4% lower caloric intake:gain 0 to 1 wk post-second dose, 16.8% lower caloric intake:gain 1 to 2 wk post-second dose, 14.3% lower caloric intake:gain 2 to 3 wk post-second dose, 16.2% lower caloric intake:gain 3 to 4 wk post-second dose, and 19.5% greater caloric intake:gain 5 to 6 wk post-second dose. Lysine intake:gain was significantly affected from 0 to 1, 4 to 5, and 5 to 6 wk post-second dose and was not significantly affected from 1 to 2, 2 to 3, and 3 to 4 wk post-second dose. Improvest males had 10.3% greater lysine intake:gain 0 to 1 wk post-second dose, 30.9% greater lysine intake:gain 4 to 5 wk post-second dose, and 43.8% greater lysine intake:gain 5 to 6 wk post-second dose.

For the weekly time intervals, there were several instances where NE significantly affected caloric intake and lysine intake; however, caloric intake:gain and lysine intake:gain was not significantly affected by NE (*P* ≥ 0.07; Table 7). These differences can be largely explained by results discussed for the post-Improvest period.

#### Overall period

There were no significant interactions between Improvest management and NE (*P* ≥ 0.27) in caloric intake, caloric intake:gain, lysine intake, or lysine intake:gain during the overall period (day 0 to marketing; Table 6). There were significant differences in caloric intake, caloric intake:gain, lysine intake, and lysine intake:gain between Improvest males and PC males during the overall period. Improvest males had lower caloric intake (3.2% lower; *P* < 0.01), lower levels of caloric intake:gain (10.2% lower; *P* < 0.01), greater lysine intake (13.4% greater; *P* < 001), and greater levels of lysine intake:gain (6.4% greater; *P* < 0.01) compared with PC males during the overall period. There were significant differences in caloric intake and lysine intake for the main effect of NE during the overall period. Pigs fed low NE diets had lower caloric intake and lysine intake compared with high NE (3.1% lower caloric intake and 3.2% lower lysine intake; *P* ≤ 0.03) during the overall period. Pigs fed medium NE diets were intermediate in their value for caloric intake and lysine intake and not different from the other two NE treatments during the overall period. Caloric intake:gain and lysine intake:gain were not affected (*P* ≥ 0.30) by the main effect of NE during the overall period.

### Carcass Traits

There were no significant interactions between Improvest management and NE (*P* ≥ 0.06) for HCW, carcass dressing percentage, backfat thickness, muscle depth, or predicted lean yield (Table 8). Numerically speaking, Improvest males were 0.3 kg lighter (*P* = 0.99) when low NE diets were fed, 2.1 kg heavier (*P* = 0.57) when medium NE diets were fed, and 1.4 kg heavier (*P* = 0.87) when high NE diets were fed (Figure 2). Overall, HCW was not different (*P* = 0.16) between Improvest males and PC males, but the numerical difference was 1.1 kg. Dressing percentage was 1.6 percentage units lower (*P* = 0.01) for Improvest males compared with PC males. Backfat thickness was 0.9 mm lower (*P* < 0.01) for Improvest males compared with PC males which resulted in a greater (*P* < 0.01) predicted lean yield for Improvest males compared with PC males (0.65 percentage unit difference).

**Table 8.** Effects of dietary NE on carcass traits for male market pigs managed with physical castration or with Improvest^1^^,^^2^

	Improvest management			NE treatment				*P* values		
	PC males	Improvest males	SEM	Low NE	Medium NE	High NE	SEM	Improvest	NE	Interaction
Hot carcass weight, kg	107.2	108.3	0.67	106.0^y^	109.0^x^	108.2^xy^	0.76	0.16	0.01	0.39
Dressing percentage, %	78.36^a^	76.76^b^	0.29	76.88^y^	78.15^x^	77.65^xy^	0.34	<0.01	0.02	0.47
Backfat thickness, mm	19.4^a^	18.5^b^	0.21	18.2^y^	19.2^x^	19.4^x^	0.26	<0.01	0.01	0.37
Muscle depth, mm	63.0	63.7	0.37	62.3^y^	63.4^xy^	64.4^x^	0.44	0.13	<0.01	0.06
Destron predicted lean yield (2023 equation)^3^, %	55.04^b^	55.69^a^	0.14	55.81^x^	55.18^y^	55.11^y^	0.17	<0.01	0.01	0.26

^a,b,x,y^Least squares means within each row of main effects with different superscripts are significantly different (*P* < 0.05).

^1^Improvest is a gonadotropin-releasing factor (GnRF) analog-diphtheria toxoid conjugate product approved for temporary suppression of testicular function in intact male pigs (Zoetis Canada Inc.); Improvest pigs received the first dose of Improvest on day 29 of the study and the second dose of Improvest on day 57 of the study; PC males = physically castrated males.

^2^Within each diet phase, diets were formulated to meet SID lysine:NE requirements for PC males or Improvest males while differing in their NE; medium NE diets were a 50:50 blend of the low NE and high NE diets mixed at the farm; low NE treatment had an average NE level of 2,212 kcal/kg, medium NE treatment had an average NE level of 2,337 kcal/kg, high NE treatment had an average NE level of 2,462 kcal/kg.

^3^The equation used for Destron predicted lean yield (2023 equation) was the following:  = 89.16298 − (1.63023 × backfat thickness) − (0.42126 × muscle depth) + (0.01930 × backfat thickness^2^) + (0.00308 × muscle depth^2^) + (0.00369 × backfat thickness × muscle depth).

Pigs fed low NE diets had 3.0 kg lighter (*P* < 0.01) HCW compared with pigs fed medium NE diets, while pigs fed high NE diets had intermediate values that were not different from the other two treatments. Pigs fed low NE diets had 1.27 percentage unit lower (*P* < 0.01) carcass dressing percentage compared with pigs fed medium NE diets, while pigs fed high NE diets had intermediate values that were not different from the other two treatments. Carcasses from pigs fed low NE diets had lower (*P* < 0.01) backfat thickness compared to carcasses from pigs fed medium and high NE diets (1.0 mm less compared with medium NE and 1.2 mm less compared with high NE) which resulted in greater (*P* < 0.01) predicted lean yield values for pigs fed low NE diets compared with pigs fed medium NE and high NE diets.

## Discussion

The basis of the current study was to compare the performance of Improvest male pigs with that of PC male pigs when fed dietary programs with differing NE, which is the most novel aspect of the study. Therefore, the discussion herein will focus on the results of the interaction between Improvest management and NE.

### Live Performance

Feed intake of grow-finish pigs is generally assumed to be closely associated with dietary energy concentration (Henry, 1985; Patience et al., 2015; Velayudhan et al., 2015). When pigs are fed diets that are low in NE, greater levels of feed intake occur as compensation to meet dietary energy requirements (Spurlock et al., 1997; Smith et al., 1999; Schinckel et al., 2012; Hong et al., 2016; Fang et al., 2019). It is generally assumed that pigs are capable of compensating in a manner in which similar caloric intake will be achieved regardless of dietary energy present in the diets, that is until either the digestive tract has reached maximum capacity or metabolic demands for energy change (Henry, 1985; Nyachoti et al., 2004; Quiniou and Noblet, 2012; Schinckel et al., 2012).

For PC males, the current study largely confirmed this assumption; caloric intake was similar among NE treatments for PC males until days 81 to 87 of the study when PC males fed the low NE diets had lower levels of caloric intake compared with PC males fed the medium NE and high NE diets. At this point in the study, it may be inferred that either the digestive tracts of PC males had reached a maximum capacity, thus inhibiting caloric intake for the PC males fed the low NE diets, or the metabolic demands for energy were altered. Additional metabolism-related studies are needed to fully understand these differences.

It was hypothesized that Improvest males would respond in a unique manner when compared with PC males, as it has been shown that appetite is greatly suppressed and consumption levels are strikingly lower for intact male pigs (pre-Improvest period in the case of Improvest males) (Knudson et al., 1985; Xue et al. 1997; Van den Broeke et al., 2016). This was certainly apparent in the current study during the pre-Improvest period as Improvest males consumed less feed (13.0% lower ADFI; *P* < 0.01) and had lower caloric intake (13.1% lower; *P* < 0.01) compared with PC males. At the same time, caloric intake was similar among the NE treatments for Improvest males during the pre-Improvest period (Improvest males—low NE = 6,125 kcal NE/d; Improvest males—medium NE = 6,192 kcal NE/d; Improvest males—high NE = 6,245 kcal NE/d; *P* > 0.12). The interesting performance aspect of managing male pigs with Improvest is the dramatic shift in feed consumption that occurs during the time when testicular function is suppressed (post-Improvest period in the case of Improvest males) (Puls et al., 2014a, 2014b; Poulsen Nautrup et al., 2018). This has been described from a mechanistic action standpoint previously by Van den Broeke et al. (2016; 2022), who stated the hypothalamic–pituitary–gonadal axis being blocked by the production of gonadotropin-releasing hormone (GnRH)-antibodies downregulates the appetite suppressing the activity of androgens and estrogens that are produced by the testes. In the current study, Improvest males consumed 13.5% less feed (and 13.5% less kcal NE) 0 to 1 wk post-second dose, 4.5% less feed (and 4.2% less kcal NE) 1 to 2 wk post-second dose, 6.6% more feed (and 6.8% more kcal NE) 2 to 3 wk post-second dose, 13.3% more feed (and 12.9% more kcal NE) 3 to 4 wk post-second dose, 14.4% more feed (and 14.2% more kcal NE) 4 to 5 wk post-second dose, and 18.2% more feed (and 18.3% more kcal NE) 5 to 6 wk post-second dose compared with PC males. It was interesting that feed intake of the Improvest males did not exceed that of the PC males until 2 to 3 wk post-second dose, largely supporting the notion that performance effects caused by the suppression of testicular function require several days to occur. During the post-Improvest period, caloric intake was similar among NE treatments for Improvest males until days 81 to 87 of the study (3 to 4 wk post-second dose) when a very interesting result was observed. Improvest males fed the low NE and medium NE diets had greater levels of caloric intake compared with Improvest males fed the high NE diets. This directly contradicted the theory that when pigs are fed diets that are low in NE, greater levels of feed intake occur as compensation to meet dietary energy requirements—the Improvest males fed low NE diets in the current study actually consumed to levels that exceeded the caloric intake of Improvest males fed high NE. However, the following week of the study (days 88 to 94; 4 to 5 wk post-second dose), Improvest males fed the low NE diets had lower levels of caloric intake compared with Improvest males fed the high NE diets, which followed the results observed for PC males for days 81 to 94 of the study and the aforementioned assumption regarding intake compensation until the point the digestive tract reaches a maximum capacity. In general, this study may provide evidence that Improvest males have a different digestive tract capacity compared with PC males that are reached at a later point in the grow-finish period. This assumption warrants future research efforts, particularly when diets low in NE and high in dietary fiber are fed to male pigs managed with Improvest.

The lack of significant interactions between Improvest management and NE for the majority of live performance traits in the current study including rate of gain and feed efficiency (particularly for those during the overall period presented in Table 4) suggests that typical response levels for male pigs managed with Improvest versus PC males can be expected when pigs are fed dietary programs with NE ranging from 2,212 kcal/kg to 2,462 kcal/kg. Similar results were reported previously by Van den Broeke et al. (2022), who also reported a lack of interactive effects between male pigs managed with Improvest and dietary programs with NE of 8.8 MJ/kg (converted 2,103 kcal/kg) or 10.2 MJ/kg (converted 2,438 kcal/kg) for finishing phase performance.

An additional matter that should be considered when formulating diets for Improvest males is the levels of amino acid intake (and particularly lysine intake) during the late finishing stages. For instance, Improvest males had 30.9% greater lysine intake:gain 4 to 5 wk post-second dose, and 43.8% greater lysine intake:gain 5 to 6 wk post-second dose. It is plausible to speculate that diets could be diluted during these production periods as a significant cost savings approach. This type of research is certainly warranted as it has been several years since lysine requirement research has been conducted for Improvest males (Boler et al., 2011; Elsbernd et al., 2017).

### Carcass Traits

The lack of significant interactions between Improvest management and NE for the carcass traits measured in the current study including HCW, carcass dressing percentage, backfat thickness, muscle depth, and predicted lean yield suggests that typical response levels for male pigs managed with Improvest versus PC males can be expected when fed dietary programs with NE ranging from 2,212 kcal/kg to 2,462 kcal/kg. Similar results were reported previously by Van den Broeke et al. (2022), who also reported a lack of interactive effects between male pigs managed with Improvest and dietary programs with NE of 8.8 MJ/kg (converted 2,103 kcal/kg) or 10.2 MJ/kg (converted 2,438 kcal/kg) for carcass weight, carcass dressing percentage, backfat thickness, muscle depth, and primal weights.

### Conclusions

Overall, typical response levels for live performance and carcass characteristics were sustained for male market pigs managed with Improvest at each of the NE treatments evaluated; however, consideration should be provided to the known production impacts of low NE diets when male market pigs are managed with Improvest. Specifically, the drastic differences in feed consumption curves for Improvest males during both the pre-Improvest (intact male phase) and post-Improvest (suppression of testicular function phase) periods when compared with PC males should be considered when feeding unique diets, such as low NE diets.

## References

[CIT0001] AOAC. 2016. Official methods of analysis of AOAC international. 20th ed. Washington DC: Association of Official Analytical Chemists, Inc

[CIT0002] Bohrer, B. M., Y. Wang, J. B. Dorleku, C. P. Campbell, and I. B. Mandell. 2023. Technical note: an update of the predicted lean yield equation for the Destron PG-100 optical grading probe. J. Anim. Sci. 101:1–8. doi:10.1093/jas/skad199 PMC10313092 37317891

[CIT0003] Boler, D. D., L. W. Kutzler, D. M. Meeuwse, V. L. King, D. R. Campion, F. K. McKeith, and J. Killefer. 2011. Effects of increasing lysine on carcass composition and cutting yields of immunologically castrated male pigs. J. Anim. Sci. 89:2189–2199. doi:10.2527/jas.2010-3640 21383034

[CIT0004] Canadian Council on Animal Care. 2009. CCAC guidelines on: the care and use of farm animals in research, teaching, and testing. Ottawa, ON: CCAC. https://ccac.ca/Documents/Standards/Guidelines/Farm_Animals.pdf

[CIT0005] Čandek-Potokar, M., M. Škrlep, and G. Zamaratskaia. 2017. Immunocastration as alternative to surgical castration in pigs. Theriogenology. 6:109–126. doi:10.5772/intechopen.68650

[CIT0006] Dunshea, F. R., J. R. D. Allison, M. Bertram, D. D. Boler, L. Brossard, R. Campbell, J. P. Crane, D. P. Hennessey, L. Huber, C. de Lange, et al. 2013. The effect of immunization against GnRF on nutrient requirements of male pigs: a review. Animal. 7:1769–1778. doi:10.1017/S1751731113001407 23931578

[CIT0007] Elsbernd, A. J., C. F. M. De Lange, K. J. Stalder, L. A. Karriker, and J. F. Patience. 2017. SID lysine requirement of immunologically and physically castrated male pigs during the grower, early and late finisher periods. J. Anim. Sci. 95:1253–1263. doi:10.2527/jas.2016.0544 28380505

[CIT0008] Fang, L. H., Y. H. Jin, S. H. Do, J. S. Hong, B. O. Kim, T. H. Han, and Y. Y. Kim. 2019. Effects of dietary energy and crude protein levels on growth performance, blood profiles, and carcass traits in growing-finishing pigs. J. Anim. Sci. Technol. 61:204–215. doi:10.5187/jast.2019.61.4.204 31452907 PMC6686147

[CIT0009] Harsh, B. N., B. Cowles, R. C. Johnson, D. S. Pollmann, A. L. Schroeder, A. C. Dilger, and D. D. Boler. 2017. A summary review of carcass cutability data comparing primal value of immunologically and physically castrated barrows. Transl. Anim. Sci. 1:77–89. doi:10.2527/tas2016.0009 32704631 PMC7205327

[CIT0010] Henry, Y. 1985. Dietary factors involved in feed intake regulation in growing pigs: a review. Livest. Prod. Sci. 12:339–354. doi:10.1016/0301-6226(85)90133-2

[CIT0011] Hong, J. S., G. I. Lee, X. H. Jin, and Y. Y. Kim. 2016. Effect of dietary energy levels and phase feeding by protein levels on growth performance, blood profiles and carcass characteristics in growing-finishing pigs. J. Anim. Sci. Technol. 58:37. doi:10.1186/s40781-016-0119-z 27795835 PMC5075758

[CIT0012] Just, A. 1982. The net energy value of balanced diets for growing pigs. Livest. Prod. Sci. 8:541–555. doi:10.1016/0301-6226(82)90032-x

[CIT0013] Knudson, B. K., M. G. Hogberg, R. A. Merkel, R. E. Allen, and W. T. Magee. 1985. Developmental comparisons of boars and barrows: I. Growth rate, carcass and muscle characteristics. J. Anim. Sci. 61:789–796. doi:10.2527/jas1985.614789x 4066537

[CIT0014] Needham, T., H. Lambrechts, and L. Hoffman. 2017. Castration of male livestock and the potential of immunocastration to improve animal welfare and production traits: invited review. S. Afr. J. Anim. Sci. 47:731–742. doi:10.4314/sajas.v47i6.1

[CIT0015] NFACC. 2014. Code of practice for care and handling of pigs. Canadian Pork Council and the National Farm Animal Care Council. https://www.nfacc.ca/codes-of-practice/pig-code

[CIT0016] Noblet, J., S. Wu, and M. Choct. 2022. Methodologies for energy evaluation of pig and poultry feeds: a review. Anim. Nutr. 8:185–203. doi:10.1016/j.aninu.2021.06.015 34977388 PMC8685914

[CIT0017] NRC. 2012. Nutritional requirements of swine. 11th rev. ed. Washington, DC: Natl. Acad. Press

[CIT0018] Nyachoti, C. M., R. T. Zijlstra, C. F. M. De Lange, and J. F. Patience. 2004. Voluntary feed intake in growing-finishing pigs: a review of the main determining factors and potential approaches for accurate predictions. Can. J. Anim. Sci. 84:549–566. doi:10.4141/a04-001

[CIT0035] Pomar and M. Marcoux, C. (2003). Comparing the Canadian pork lean yields and grading indexes predicted from grading methods based on Destron and Hennessy probe measurements. Can. J. Anim. Sci. 83(3): 451-458. doi:10.4141/A02-107

[CIT0019] Patience, J. F., M. C. Rossoni-Serão, and N. A. Gutiérrez. 2015. A review of feed efficiency in swine: biology and application. J. Animal. Sci. Biotechnol. 6:1–9. doi:10.1186/s40104-015-0031-2 PMC4527244 26251721

[CIT0020] PIC. 2021. PIC® nutrition and feeding guidelines. PIC Genetics. https://www.pic.com/resources

[CIT0021] Poulsen Nautrup, B., I. Van Vlaenderen, A. Aldaz, and C. K. Mah. 2018. The effect of immunization against gonadotropin-releasing factor on growth performance, carcass characteristics and boar taint relevant to pig producers and the pork packing industry: a meta-analysis. Res. Vet. Sci. 119:182–195. doi:10.1016/j.rvsc.2018.06.002 29958153

[CIT0022] Puls, C. L., M. Ellis, F. K. McKeith, A. M. Gaines, and A. L. Schroeder. 2014a. Effects of ractopamine on growth performance and carcass characteristics of immunologically and physically castrated barrows and gilts. J. Anim. Sci. 92:4725–4732. doi:10.2527/jas.2014-7882 25149340

[CIT0023] Puls, C. L., A. Rojo, M. Ellis, D. D. Boler, F. K. McKeith, J. Killefer, A. M. Gaines, P. D. Matzat, and A. L. Schroeder. 2014b. Growth performance of immunologically castrated (with Improvest) barrows (with or without ractopamine) compared to gilt, physically castrated barrow, and intact male pigs. J. Anim. Sci. 92:2289–2295. doi:10.2527/jas.2013-6861 24671576

[CIT0024] Quiniou, N., and J. Noblet. 2012. Effect of the dietary net energy concentration on feed intake and performance of growing-finishing pigs housed individually. J. Anim. Sci. 90:4362–4372. doi:10.2527/jas.2011-4004 22696619

[CIT0025] Rueff, L., M. A. Mellencamp, and L. Galina Pantoja. 2019. Performance of immunologically castrated pigs at a commercial demonstration farm over 3.5 years. J. Swine Health Prod. 27:322–328. https://www.aasv.org/shap/issues/v27n6/v27n6p322.pdf.

[CIT0026] Schinckel, A. P., M. E. Einstein, S. Jungst, J. O. Matthews, C. Booher, T. Dreadin, C. Fralick, E. Wilson, and R. D. Boyd. 2012. Daily feed intake, energy intake, growth rate and measures of dietary energy efficiency of pigs from four sire lines fed diets with high or low metabolizable and net energy concentrations. Asian-Australas. J. Anim. Sci. 25:410–420. doi:10.5713/ajas.2011.11212 25049580 PMC4092956

[CIT0027] Smith, J. W., M. D. Tokach, P. R. O’Quinn, J. L. Nelssen, and R. D. Goodband. 1999. Effects of dietary energy density and lysine: calorie ratio on growth performance and carcass characteristics of growing-finishing pigs. J. Anim. Sci. 77:3007–3015. doi:10.2527/1999.77113007x 10568471

[CIT0028] Spurlock, M. E., G. R. Frank, G. M. Willis, J. L. Kuske, and S. G. Cornelius. 1997. Effect of dietary energy source and immunological challenge on growth performance and immunological variables in growing pigs. J. Anim. Sci. 75:720–726. doi:10.2527/1997.753720x 9078489

[CIT0029] Van den Broeke, A., M. Aluwé, K. Kress, V. Stefanski, M. Škrlep, N. Batorek, B. Ampe, and S. Millet. 2022. Effect of dietary energy level in finishing phase on performance, carcass and meat quality in immunocastrates and barrows in comparison with gilts and entire male pigs. Animal. 16:100437. doi:10.1016/j.animal.2021.100437 35007882

[CIT0030] Van den Broeke, A., F. Leen, M. Aluwé, B. Ampe, J. Van Meensel, and S. Millet. 2016. The effect of GnRH vaccination on performance, carcass, and meat quality and hormonal regulation in boars, barrows, and gilts. J. Anim. Sci. 94:2811–2820. doi:10.2527/jas.2015-0173 27482668

[CIT0031] Velayudhan, D. E., I. H. Kim, and C. M. Nyachoti. 2015. Invited review - characterization of dietary energy in swine feed and feed ingredients: a review of recent research results. Asian-Australas. J. Anim. Sci. 28:1–13. doi:10.5713/ajas.14.0001R 25557670 PMC4283177

[CIT0032] Woyengo, T. A., E. Beltranena, and R. T. Zijlstra. 2014. Nonruminant nutrition symposium: controlling feed cost by including alternative ingredients into pig diets: a review. J. Anim. Sci. 92:1293–1305. doi:10.2527/jas.2013-7169 24492540

[CIT0033] Xue, J. L., G. D. Dial, and J. E. Pettigrew. 1997. Performance, carcass, and meat quality advantages of boars over barrows: a literature review. Swine Health Prod. 5:21–28. https://www.aasv.org/shap/issues/v5n1/v5n1p21.pdf.

[CIT0034] Zuidhof, M. 2019. A review of dietary metabolizable and net energy: uncoupling heat production and retained energy. J. Appl. Poult. Res. 28:231–241. doi:10.3382/japr/pfx062

